# Transmission to Interneurons Is via Slow Excitatory Synaptic Potentials Mediated by P2Y_1_ Receptors during Descending Inhibition in Guinea-Pig Ileum

**DOI:** 10.1371/journal.pone.0040840

**Published:** 2013-02-07

**Authors:** Peter D. J. Thornton, Rachel M. Gwynne, Darren J. McMillan, Joel C. Bornstein

**Affiliations:** Department of Physiology, University of Melbourne, Parkville, Victoria, Australia; University of Chicago, United States of America

## Abstract

**Background:**

The nature of synaptic transmission at functionally distinct synapses in intestinal reflex pathways has not been fully identified. In this study, we investigated whether transmission between interneurons in the descending inhibitory pathway is mediated by a purine acting at P2Y receptors to produce slow excitatory synaptic potentials (EPSPs).

**Methodology/Principal findings:**

Myenteric neurons from guinea-pig ileum *in vitro* were impaled with intracellular microelectrodes. Responses to distension 15 mm oral to the recording site, in a separately perfused stimulation chamber and to electrical stimulation of local nerve trunks were recorded. A subset of neurons, previously identified as nitric oxide synthase immunoreactive descending interneurons, responded to both stimuli with slow EPSPs that were reversibly abolished by a high concentration of PPADS (30 μM, P2 receptor antagonist). When added to the central chamber of a three chambered organ bath, PPADS concentration-dependently depressed transmission through that chamber of descending inhibitory reflexes, measured as inhibitory junction potentials in the circular muscle of the anal chamber. Reflexes evoked by distension in the central chamber were unaffected. A similar depression of transmission was seen when the specific P2Y_1_ receptor antagonist MRS 2179 (10 μM) was in the central chamber. Blocking either nicotinic receptors (hexamethonium 200 μM) or 5-HT_3_ receptors (granisetron 1 μM) together with P2 receptors had no greater effect than blocking P2 receptors alone.

**Conclusions/Significance:**

Slow EPSPs mediated by P2Y_1_ receptors, play a primary role in transmission between descending interneurons of the inhibitory reflexes in the guinea-pig ileum. This is the first demonstration for a primary role of excitatory metabotropic receptors in physiological transmission at a functionally identified synapse.

## Introduction

It has been more than a century since Bayliss and Starling formulated the “Law of the Intestine” to account for the intestinal reflexes that underlie intestinal propulsion [1]. During this time, there have been many studies designed to identify the neural circuitry responsible for review see [2]. The two fundamental reflexes identified by these pioneers are ascending excitation and descending inhibition, each of which is evoked by local stimulation of an intestinal segment and which they demonstrated are mediated by the gut's intrinsic nervous system, the enteric nervous system. Later studies confirmed this and identified other reflexes triggered by related stimuli including reflex accommodation [3] and a descending excitatory reflex [4]. Stimuli that evoke these reflexes include distension of the intestinal wall, mucosal deformation and local application of physiologically relevant chemicals like acid and amino acids (e.g. [5], [6]). However, key questions about their interconnections and the dynamic properties of the circuits remain unresolved.

Over the last 3 decades, the enteric neurons that control intestinal motor behaviour in guinea-pig ileum have been identified via immunohistochemical, morphological and electrophysiological studies allowing a static model of the enteric circuits to be constructed (for review see [2]). These circuits include sensory neurons intrinsic to the gut wall, several classes of interneurons and both excitatory and inhibitory motor neurons. The axonal projections of interneurons and motor neurons are polarized, so that orally directed (ascending) and anally directed (descending) pathways can be identified. However, the axons of each pathway run in nerve trunks common to all so that electrical stimuli cannot be reliably used to activate specific subclasses of enteric neurons.

To overcome the problem of non-specific activation of pathways by electrical stimulation, analysis of synaptic transmission and the connectivity between individual classes of neurons has largely come from studies of functional responses evoked by physiological stimuli like distension, mucosal deformation or chemical stimuli applied to the mucosa [5], [7] (for review see [8]). When used in combination with multichambered organ baths that allow specific antagonists to be applied to a restricted part of each reflex pathway, these methods have identified transmitter receptor combinations that are involved at some of the key synapses. For example, transmission between interneurons in the ascending excitatory pathway of the guinea-pig small intestine appears to be exclusively via nicotinic acetylcholine receptors [9]. Transmission from intrinsic sensory neurons to ascending interneurons and from ascending interneurons to excitatory motor neurons requires both nicotinic acetylcholine receptors and NK_3_ tachykinin receptors [10]. The picture is murkier for the descending reflex pathways of the small intestine. While transmission from interneurons to inhibitory motor neurons apparently depends exclusively on activation of P2X receptors [11], no transmitter or receptor has been identified at other synapses in the descending inhibitory pathway. Indeed, antagonists to all transmitter/receptor combinations that mediate fast excitatory synaptic potentials (EPSPs) in the enteric nervous system have no effect either individually, or in combination, on transmission of reflex activity between intrinsic sensory neurons and either interneurons or inhibitory motor neurons or on transmission between interneurons [11]. Two lines of evidence suggest that part of the answer may be that transmission between descending interneurons in the inhibitory pathway may be via slow EPSPs mediated by P2Y_1_ purine receptors. Using a two chambered organ bath and recording from myenteric neurons in the anal chamber, we found that distension evokes slow EPSPs in a subset of neurons identified by morphology and immunohistochemistry as descending interneurons immunoreactive for nitric oxide synthase (NOS) [12]. Furthermore, these slow EPSPs were found to be the result of synaptic transmission from descending interneurons with cell bodies close to the distension sensitive sensory neurons. In a second series of experiments, we showed that individual electrical stimuli applied focally to the mucosa evoked P2Y_1_ mediated slow EPSPs in the same population of descending interneurons [13]. The question remains whether the slow EPSPs evoked by distension are mediated by P2Y receptors and if blocking these receptors will modify descending inhibition. This study was designed to test these questions using intracellular recording from myenteric neurons and recording from circular smooth muscle of guinea-pig ileum.

We found that the distension-evoked slow EPSPs in NOS-immunoreactive descending interneurons were mediated by P2Y receptors and that blockade of this transmission between interneurons depressed descending inhibitory reflexes evoked by the same stimulus. Thus, a substantial component of excitatory transmission in this well defined reflex pathway is mediated not by conventional ionotropic receptors and fast EPSPs, but via a metabotropic receptor and a much slower EPSP. To our knowledge this is the first demonstration of physiological transmission via an excitatory metabotropic receptor raising the possibility that similar mechanisms may operate at other synapses including in the brain.

## Results

### Studies of myenteric neurons that respond to distension

**Figure 1:**
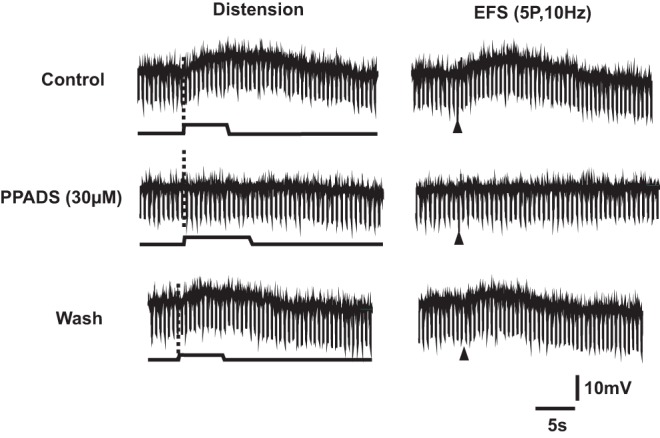
PPADS (30 μM) blocks distension and electrically evoked slow EPSPs in a NOS-immunoreactive descending interneuron. Left column shows responses to distension: the upper trace in each pair is the membrane potential, the lower trace is the time course of the applied distension. Downward deflections show the membrane potential changes produced by constant amplitude current pulses used to monitor any changes in input resistance. The right column shows responses to focal electrical stimulation (5 pulses at 10 Hz, arrow heads) applied to a circumferentially directed internodal strand. The dashed lines indicate a fast EPSP evoked by a single impulse in the train (shown with expanded time base below). The evoked slow depolarizations evoked by either stimulus were reversibly abolished by 30 μM PPADS in the recording chamber within 10 minutes of exposure.

**Figure 2:**
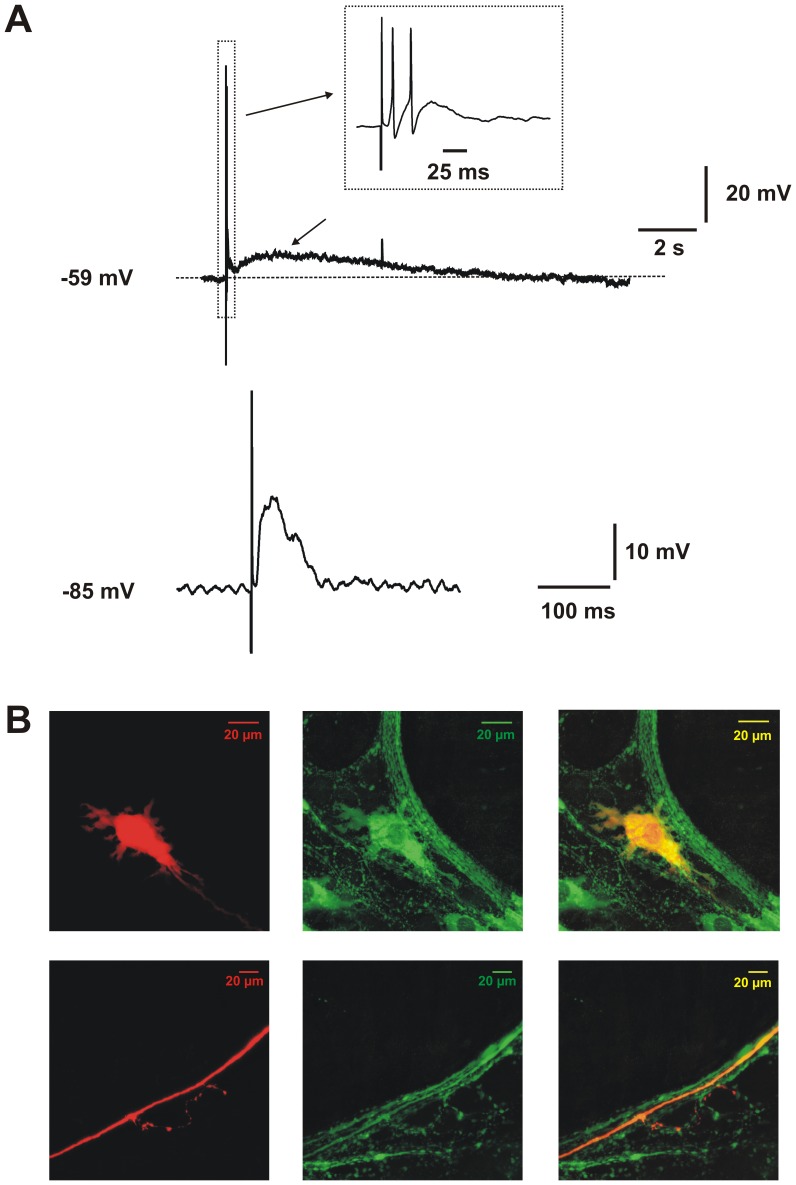
Fast and slow EPSPs in a nitric oxide synthase immunoreactive descending interneuron. A Synaptic potentials evoked by electrical stimulation of the mucosa. Upper trace: membrane potential response at resting membrane potential, stimulus applied to neighbouring mucosa. Inset shows the EPSP evoked with superimposed action potentials on an expanded time base (arrows). Lower trace: fast EPSP (arrow) evoked by the same stimulus when the membrane potential was hyperpolarized to prevent action potential firing and increase the driving potential for the inward current. B Left panels: the impaled neuron stained to reveal biocytin (red) in the cell body (upper) and part of its axon with a side-branch providing varicose endings in a more anal ganglion (lower). Centre panels: nNOS-immunoreactivity (green) of the same fields. Right panels: superimposed images showing that the impaled neuron and its processes are nNOS-immunoreactive (yellow/orange).

In all, 43 S neurons were identified as suitable for further investigation of responses to distention as they responded to distension with a burst of fast excitatory synaptic potentials (EPSPs) and/or a slow EPSP. Slow EPSPs were seen in 13 neurons in response to distension applied in the oral chamber of a two chambered organ bath (Figure 1). Fast EPSPs evoked by distension in these neurons were typically very small (1–2 mV at the resting membrane potential of between −55 mV and −45 mV) and were not seen at all in 5 neurons with slow EPSPs (Figure 1 left column). To quantify the relative number of neurons with both fast and slow EPSPs evoked by distension, we reanalyzed the data used in our previous study [12], thereby increasing the sample size to 29 neurons with slow EPSPs evoked by distension. Distension did not evoke fast EPSPs in 7 of these neurons, but all 29 exhibited robust fast EPSPs (Figure 2A) in response to focal stimulation of circumferentially directed intermodal strands. In most cases, fast EPSP responses to distension were intermittent with only 5 neurons exhibiting such synaptic potentials in response to every distension (typically 3–4 distensions per neurons), although slow EPSPs were consistently evoked by the same stimulus. In 19 of the 22 neurons with distension-evoked slow and fast EPSPs, the amplitudes of the slow EPSPs (range 3.0–7.1 mV) were larger than those of the distension-evoked fast EPSPs (range 1.3–3.1 mV) at the resting membrane potential of about −50 mV. In one neuron, fast EPSPs evoked by distension ranged from 2.2 mV to 18.5 mV with many triggering an action potential, while the mean slow EPSP was 8.3±1.6 mV (4 distensions). In two other neurons, the distension evoked fast and slow EPSPs had similar amplitudes, but the fast EPSPs were few and intermittent. Thus, distension predominantly evoked slow EPSPs in this subset of neurons and, as analysis of the functions of the slow EPSPs was the predominant goal of this study, these neurons are termed distension-responsive neurons below.

Focal electrical stimulation (5 pulses, 10 Hz) of a circumferentially directed internodal strand evoked slow EPSPs in all 10 distension-responsive neurons tested (Figure 1 right column). A similar stimulus evoked slow EPSPs in a much smaller proportion of neurons that did not respond to distension with a slow EPSP (2 of 22 tested; *P*<0.001 χ^2^ test, 1 d.f.).

The latencies, rise times, amplitudes and durations of the slow EPSPs evoked by distension and by electrical stimulation in the distension-responsive neurons were similar to those reported previously [12]. Only the amplitudes differed between the two types of slow EPSP, with those evoked by distension being significantly smaller (distension 4.1±0.5 mV, electrical stimulation 7.8±1.2 mV; *P*<0.005, t test).

Of the 13 distension-responsive neurons, 9 were well enough filled with biocytin to be identified after fixation and processing to reveal the morphology of their cell bodies. All 9 had relatively large cell bodies, lamellar dendrites and a single axon that projected anally. In 8 cases, the axon could be followed through several ganglia before either fading or reaching the end of the preparation. In 7 of these cells, the axon produced varicose side branches in one or more ganglia (Figure 2B), indicating that it was an interneuron [12], [13]. Immunoreactivity for NOS was examined for 6 of these neurons and all were immunoreactive for this enzyme (Figure 2B). These observations are consistent with our earlier conclusion that distension evokes slow EPSPs in NOS-immunoreactive descending interneurons, but not in other subclasses of neurons in descending reflex pathways [12].

The P2 receptor antagonist PPADS (30 μM) added to the recording chamber blocked the slow EPSPs evoked by distension (n = 4) and those evoked by electrical stimulation (n = 3) in distension-responsive neurons (Fig. 1). PPADS was chosen for this experiment, rather than more specific antagonists acting at P2Y_1_ receptors, because we intended to use it in studies of reflexes evoked in the circular muscle and 10 µM PPADS blocks P2X mediated fast EPSPs. Thus, it was possible to compare the effects of blocking fast and slow EPSPs mediated by purines using a single concentration-effect relationship. Our previous results had indicated that both PPADS and the more selective P2Y_1_ antagonist MRS2179 abolished slow EPSPs evoked in NOS-immunoreactive descending interneurons by electrical stimulation of the mucosa [13]. Note, abolition of the distension-evoked slow EPSP did not reveal significant fast EPSPs in response to distension (Fig. 1 left column). PPADS (30 μM) also blocked the electrically evoked slow EPSP in one neuron that lacked a slow EPSP in response to distension.

### Specificity of PPADS

The observations above indicate that 30 µM PPADS is a useful tool to identify the role of slow EPSPs evoked by distension, but this depends on its selectivity for P2Y receptors, compared with other receptors known to produce slow EPSPs, of this high concentration of the antagonist. We have shown that this concentration of PPADS does not depress slow EPSPs mediated by NK_1_ tachykinin receptors evoked in AH-neurons by mucosal stimulation [13]. However, to confirm the selectivity of 30 μM PPADS, additional experiments were undertaken.

**Figure 3:**
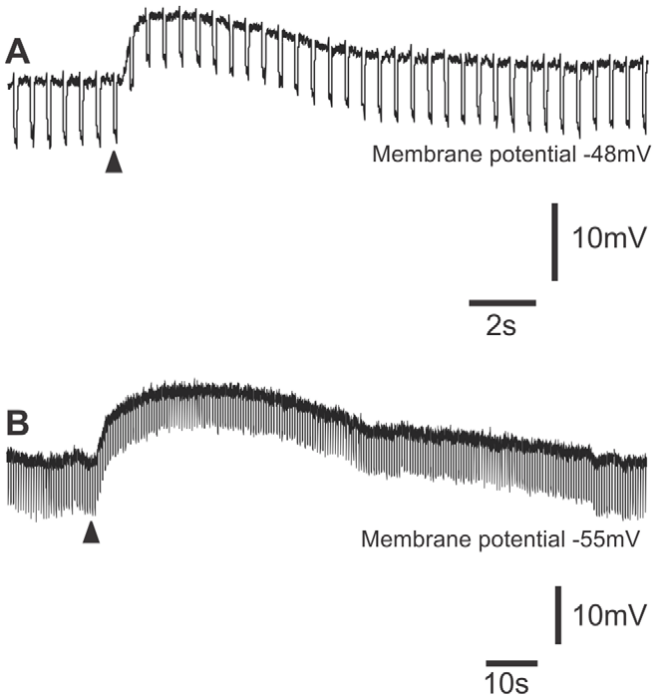
Effects of local application of ATP to myenteric S neurons. A. Application of ATP (arrowhead) evoked a rapid depolarization with a prominent decline in the membrane potential change evoked by hyperpolarizing current pulses, i.e. an increase in membrane conductance. B. Application of ATP (arrowhead) evoked a prolonged depolarization with no apparent change in membrane conductance in a distension-responsive neuron. Note the different time bases.

Local application of ATP (100 µM in HEPES buffered saline, pH 7.4) from a pressure pipette depolarized 33 of the 42 S-neurons studied, as has been described previously [14]. In 13 of these neurons, the depolarization was relatively rapid (time to peak 200 ms –2 s, duration 8–10 s) and associated with a reduction in input resistance (Figure 3A). This was probably due to activation of P2X receptors. In 7 neurons, the depolarization was much slower (rising phase up to 15 s, duration up to 150 s) and with little or no change in input resistance (Figure 3B). In the remaining 13 neurons, both types of responses to ATP were observed. As expected, 30 µM PPADS (which should block both P2X and P2Y_1_ receptors) blocked these depolarizations in all neurons tested including 5 distension-responsive neurons (not illustrated). Thus, ATP mimics the slow EPSPs in the distension-responsive neurons and is blocked by the same antagonist.

PPADS (30 µM) was ineffective against slow EPSPs evoked in AH-neurons by stimulation of circumferentially directed internodal strands in cleared myenteric plexus preparations (not illustrated). These slow EPSPs are blocked by NK_1_ and NK_3_ tachykinin receptor antagonists [15], [16]. Thus, PPADS does not block slow EPSPs evoked by tachykinins in myenteric neurons, a result consistent with the finding that PPADS does not block slow EPSPs evoked in AH-neurons by electrical stimulation of the mucosa [13].

Electrical stimulation of descending pathways in the myenteric plexus can excite slow EPSPs in AH-neurons that are blocked by the 5-HT_7_ antagonist SB 269970 indicating that they are mediated by serotonin acting at 5-HT_7_ receptors [17]. However, 30 µM PPADS had no effect on depolarizations evoked by focal application of serotonin in any neuron tested (5 AH-neurons, 1 S-neuron; not illustrated). Thus, effects of PPADS are unlikely to be via blockade of 5-HT receptors.

### Descending Inhibitory Reflexes

**Figure 4:**
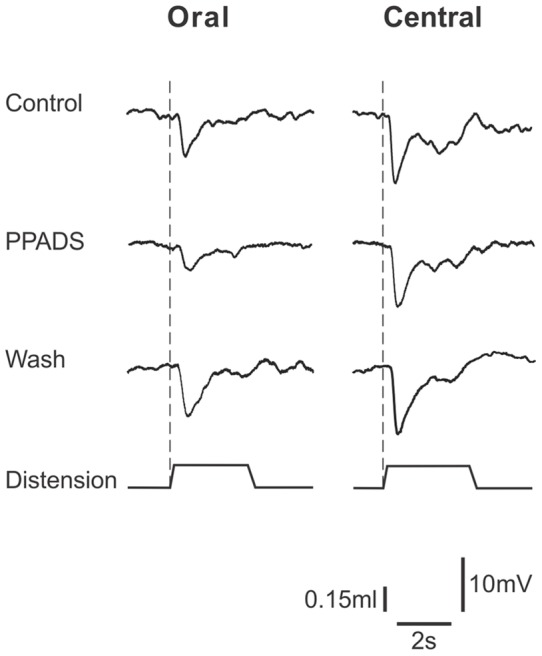
PPADS (30 μM) depresses descending inhibitory reflexes by an action on synapses between interneurons. The left column shows IJPs evoked in circular muscle in the anal chamber by distension (time course shown in lower trace of each panel) applied in the oral (far) chamber of a three chambered organ bath. IJPs were depressed when PPADS (30 µM) was present in the central chamber of this organ bath and this was partially reversed on washout of the P2 antagonist. The right column shows IJPs evoked in the anal recording chamber by distension applied in the central chamber. PPADS had no effect. As PPADS can only act on synapses in the central chamber under these conditions, the depression of transmission through the central chamber is likely to be due to an action on transmission between interneurons, while transmission from the distension-sensitive sensory neurons was unaffected.

**Figure 5:**
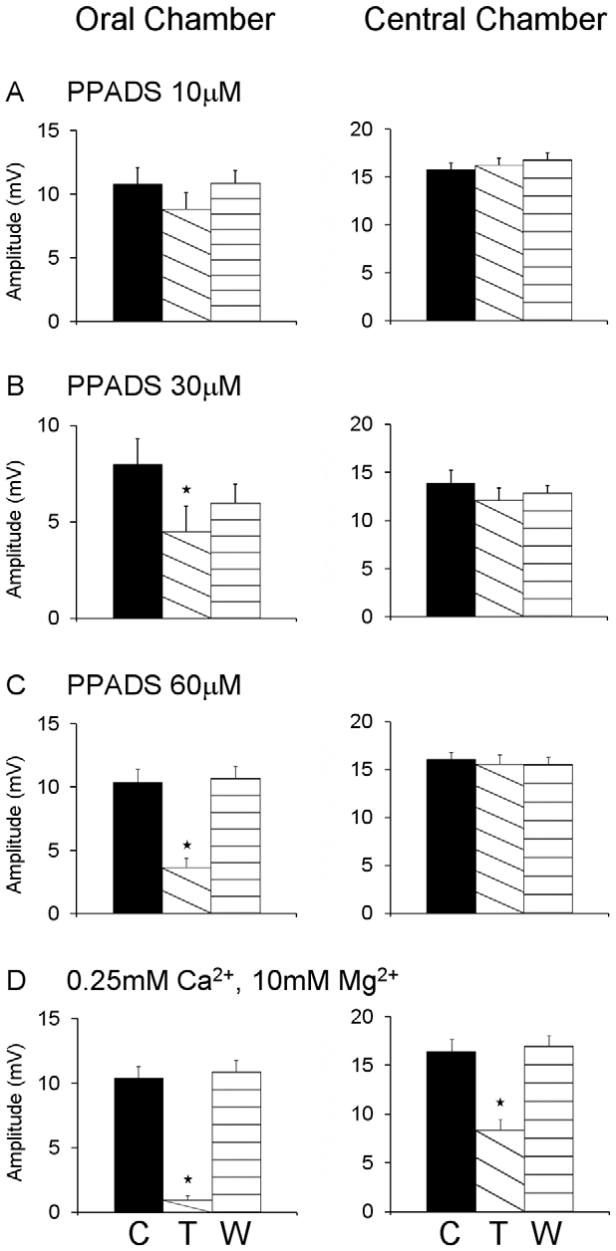
Concentration-effect relation for PPADS in the central chamber on descending inhibitory reflexes. The effects of 10 µM (top panels), 30 µM (second panels) and 60 µM (third panels) PPADS in the central chamber on IJPs evoked in the anal chamber by distension in the far (oral) chamber (left column) and central chambers (n≥6 in each case). These are compared with the effects of complete synaptic blockade by a 0.25 mM Ca^2+^, 10 mM Mg^2+^ solution in the central chamber on the same responses (n = 6). Synaptic blockade significantly depressed reflexes whether they were evoked in the far or the central chamber, although it was much more effective on reflexes conducted through this chamber than those evoked in it. The effect of PPADS on reflexes conducted through the central chamber from far chamber distension was concentration-dependent with 10 µM having no effect, but even 60 µM PPADS was less effective than complete synaptic blockade. C indicates data obtained in control physiological saline, D indicates data obtained with either PPADS or the low Ca^2+^, high Mg^2+^ solution in the central chamber, W indicates data obtained at least 20 minutes after the solution in the central chamber was returned to control.

As has been reported previously, distensions applied in either the oral or the central chamber of a three chambered organ bath evoked robust inhibitory junction potentials (IJPs) in the circular muscle when recording in the anal chamber of that bath [11]. PPADS added to the central chamber produced a reversible concentration-dependent depression of the amplitudes of the IJPs evoked in the recording chamber by distension applied in the oral chamber. The concentration (30 µM) that abolished distension-evoked slow EPSPs reduced the IJPs by 43% (control 8.3±0.8 mV, PPADS 4.7±0.7 mV, washout 6.8±0.5 mV; *P*<0.0001 ANOVA) (Figures 4 and 5). At 10 μM, PPADS in the central chamber had no significant effect on IJPs evoked by oral chamber distension (*P*>0.05), while 60 μM PPADS in the central chamber reversibly reduced IJPs evoked by oral chamber distension by 65% (*P*<0.0001) (Figure 5). Thus, PPADS reduced transmission of the descending inhibitory reflex through the central chamber. By contrast, PPADS in the central chamber had no significant effect on IJPs evoked by stimuli applied in this chamber at any of the concentrations tested.

The effects of PPADS were compared with the effects of blocking all synaptic transmission in the central chamber with a low Ca^2+^ (0.25 mM), high Mg^2+^ (12 mM) solution (Figures 5D) [9], [10], [11]. This solution reduced the amplitude of IJPs evoked in the recording chamber by distension applied in the oral chamber from 10.4±1.0 mV to 1.0±0.3 mV (90%, *P*<0.0001) and IJPs evoked by distensions applied in the central chamber from 16.4±1.3 mV to 8.4±1.2 mV (49%, *P*<0.0001). These reductions were substantially greater than the effects of PPADS (30–60 μM).

**Figure 6:**
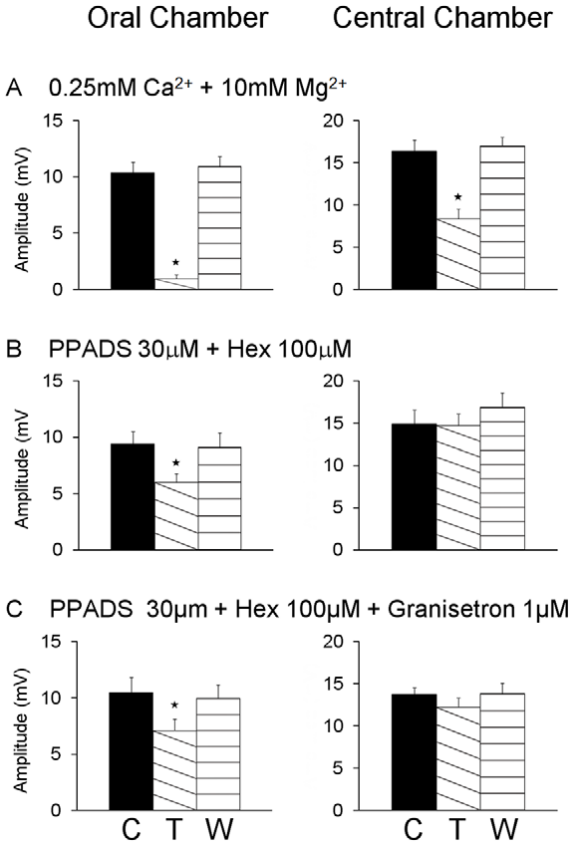
Lack of additional effect of blocking nicotinic acetylcholine receptors and 5-HT_3_ receptors together with P2 receptors. Top panels show the effects of 30 µM PPADS in the central chamber on reflexes conducted through that chamber (Far chamber distension, left) and reflexes evoked in that chamber (Central chamber distension, right). Data shown in these panels is also shown in Figure 5. Addition of the nicotinic receptor antagonist, hexamethonium (100 µM, second from top panels) or both hexamethonium and the 5-HT_3_ receptor antagonist, granisetron (1 µM, third from top panels) together with PPADS had no greater effect than PPADS alone. The bottom panels show the effects of complete synaptic blockade in the central chamber for comparison. C – control, D – antagonists present in central chamber, W – after 20 minutes of washout of antagonists.

In an attempt to identify the nature of the PPADS-resistant transmission in the central chamber, hexamethonium (100 μM) was added together with PPADS (30 μM) to the central chamber. The effect on descending inhibitory reflexes was no greater than that of PPADS alone (Figure 6 A, B). IJPs evoked by distension in the oral stimulation chamber were reduced by 34% (*P*<0.002), but this was actually less than the reduction produced by PPADS alone. IJPs evoked by distension in the central chamber were unaffected by this combination of antagonists.

The addition of the 5-HT_3_ antagonist granisetron (1 μM), together with PPADS (30 μM) and hexamethonium (100 μM), to the central chamber also had no greater effect (32%) than that of PPADS alone (Figure 6 A, C). PPADS, hexamethonium and granisetron together in the central chamber had no effect on IJPs evoked by distensions applied in this chamber.

**Figure 7:**
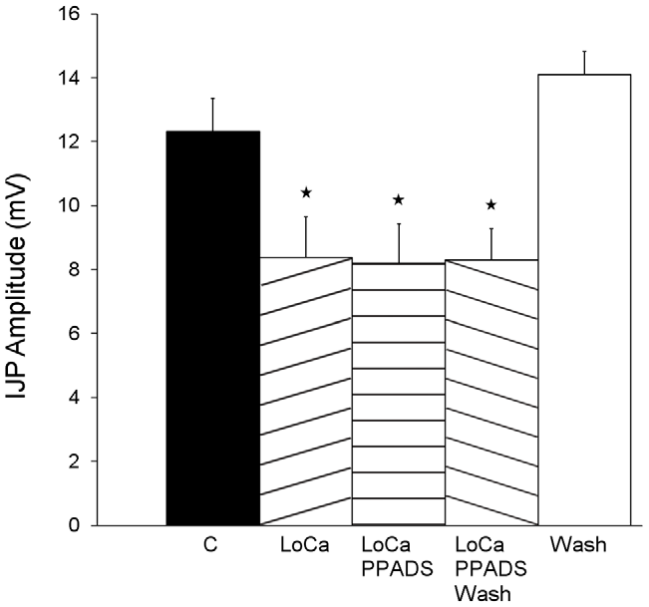
PPADS does not affect transmission from distension-activated sensory neurons to inhibitory motor neurons. Histograms showing that blocking P2 receptors in the recording chamber does not alter the inhibitory response evoked by distension in the central chamber when synaptic transmission in the central chamber is blocked with a 0.25 mM Ca^2+^, 10 mM Mg^2+^ solution. C – control IJPs; LoCa –0.25 mM Ca^2+^, 10 mM Mg^2+^ in the central stimulation chamber; LoCa PPADS – as for LoCa, but with 30 µM PPADS in the recording chamber; LoCa PPADS Wash – after washout of PPADS from the recording chamber; LoCa Wash – after return to control solutions in the central chamber. * significantly different (P<0.05) from the original control.

We examined whether P2Y-mediated transmission has a role in transmission between the descending axons of distension-sensitive intrinsic sensory neurons and inhibitory motor neurons. Synaptic transmission was blocked in the central chamber using the low Ca^2+^, high Mg^2+^ solution and the effects of 30 μM PPADS added to the recording chamber on IJPs evoked by distensions applied in the central chamber were examined (Figure 7). The low Ca^2+^, high Mg^2+^ solution depressed the IJPs from 12.3±1.0 mV to 8.4±1.3 mV (32% reduction, *P*<0.0001), but adding PPADS to the recording chamber had no further effect (8.2±1.2 mV; *P*>0.05).

The effect of adding the selective P2Y_1_ receptor antagonist MRS 2179 (10 μM) to the central chamber of a three chambered organ bath was studied using extracellular suction electrodes to record the IJPs evoked by distension. The antagonist significantly depressed the IJPs evoked by distension applied in the far stimulation chamber by 28% (*P*<0.05, not illustrated). This confirmed that at least part of the effect of PPADS was due to blockade of P2Y_1_ receptors.

## Discussion

This study provides strong evidence that slow EPSPs mediated by metabotropic P2Y_1_ purinoceptors play the primary role in transmission between interneurons of the descending inhibitory pathway of the guinea-pig ileum. This is the most direct evidence to date that excitatory metabotropic receptors, not only modulate transmission via ionotropic receptors, but actually produce physiologically meaningful EPSPs. The data also indicate that transmission from distension-sensitive intrinsic sensory neurons to other neurons of the descending inhibitory pathway is not mediated by P2Y_1_ receptors, nicotinic acetylcholine receptors, P2X receptors or 5-HT_3_ receptors.

### P2Y_1_ receptors mediate slow EPSPs evoked by distension

Distension at the oral end of isolated guinea-pig ileum evokes slow EPSPs exclusively in anally located myenteric neurons identified immunohistochemically and morphologically as NOS-immunoreactive descending interneurons [12]. This earlier study also showed that blockade of synaptic transmission in the stimulation chamber abolished distension evoked slow EPSPs indicating that they arise at synapses between descending interneurons. The present study shows that slow EPSPs are the major form of excitation evoked by distension in these neurons with the fast EPSPs evoked by this stimulus often being intermittent and small (1–2 mV at resting membrane potential) or even absent under these experimental conditions (see Figures 2, 3). Electrical stimuli applied to intermodal strands or to the intestinal mucosa evoke robust fast EPSPs in these neurons (e.g. Fig. 1 and [13], so they receive such inputs, but distension is not effective in exciting them. Individual stimuli applied to the mucosa also evoke P2Y_1_ mediated (blocked by PPADS and MRS2179) slow EPSPs in these neurons [13]. Thus, stimuli applied to neighboring mucosa (1–2 mm from the impaled neuron), to an interganglionic connective (100–250 µm from the neuron) or via distension 2 cm from the impaled neuron, all evoke slow EPSPs that are abolished by PPADS at a concentration that blocks P2Y receptors. Thus, we conclude that all slow EPSPs in these neurons are mediated by P2Y receptors, probably P2Y_1_ receptors.

This effect of PPADS was almost certainly due to a direct action on the NOS-neurons, rather than elsewhere in the pathway, because PPADS was applied only in the recording chamber. Further, neurons were impaled within 2 rows of ganglia of the chamber divider, so it is unlikely that there was an intervening synapse between the site of action of PPADS and the NOS-interneurons. That PPADS blocked the depolarizations evoked in these neurons by directly appliedof ATP further strengthens this conclusion.

### Slow EPSPs evoked via P2Y_1_ receptors have a primary role in synaptic transmission between interneurons of the descending inhibitory pathway

The finding that PPADS abolishes distension-evoked slow EPSPs in NOS-immunoreactive descending interneurons gave us the ideal opportunity to examine the physiological roles of these EPSPs and the functions of NOS-interneurons. Our multi-chambered organ bath system allowed distensions to be delivered in the oral or central compartments, while recordings were made from the circular smooth muscle in the anal compartment. Each chamber was separately perfused so that antagonists were applied to different parts of reflex pathways excited by distension [10]. Thus, transmission between functionally defined neurons was studied without the ambiguity inherent when antagonists can act anywhere along a reflex pathway [8]. PPADS, or MRS2179, in the central chamber depressed descending inhibitory reflexes evoked by distension in the oral chamber indicating that P2 purinoceptors mediate transmission of activity along this reflex pathway. However, PPADS in the central chamber had no effect on inhibitory reflexes evoked by distension applied in this chamber indicating that P2 receptors do not mediate either mechanosensory transduction of distension or transmission from distension-sensitive intrinsic sensory neurons to interneurons or to the long inhibitory motor neurons seen in this preparation [18].

Descending inhibition is not the only anally directed reflex in guinea pig ileum. When the smooth muscle is free to contract, descending excitation is the predominant reflex response to distension [4], [19]. This is abolished when L-type Ca^2+^ channels are blocked in the stimulation chamber of a three chambered organ bath [20]. Several mechanisms may account for this including an increased threshold for reflex activation due to loss of muscle tone, loss of feedback from the mucosa due to inhibition of contraction induced serotonin release [21] or a reduction in the sensitivity of distension activated intrinsic sensory neurons to sustained stretch [22]. In our study, the smooth muscle was paralyzed by nicardipine (L-type Ca^2+^ channel blocker) and excitatory neuromuscular transmission depressed by hyoscine, conditions that spare descending inhibition. Thus, the distension evoked synaptic potentials in this study are a subset of those evoked when the smooth muscle can contract normally and are probably specifically involved in descending inhibition. The relative contribution of slow EPSPs in interneurons to the entire reflex response to distension cannot be determined from the present analysis.

That 10 µM PPADS in the central chamber did not significantly depress transmission through this chamber is consistent with earlier conclusions that P2X receptors are not involved in transmission between descending interneurons in the descending inhibitory pathway [11], [23]. This concentration of PPADS abolishes fast EPSPs mediated by P2X receptors in guinea-pig myenteric neurons [24] and blocks descending excitation suggesting involvement of P2X receptors in this excitatory reflex [19], [25]. These data also suggest that transmission from descending interneurons or distension sensitive intrinsic sensory neurons to long inhibitory motor neurons is not mediated by P2X receptors, a conclusion implicit in the earlier studies.

At higher concentrations, PPADS significantly depressed transmission through the central chamber, which suggests P2Y receptor mediated transmission to descending interneurons and/or long inhibitory motor neurons. As PPADS-sensitive slow EPSPs are predominantly in NOS-immunoreactive descending interneurons (see also [12], [13], it is highly probable that transmission to these neurons from more orally placed interneurons is depressed in the reflex studies.

PPADS was much less effective in blocking transmission through the central chamber than low Ca^2+^, high Mg^2+^, although 30–60 µM PPADS should abolish slow EPSPs in NOS interneurons. However, there are parallel descending inhibitory pathways: one carried via interneurons and the other via long anally directed axons of distension sensitive intrinsic sensory neurons [10], [11]. PPADS will block the former, but not the latter. Indeed, 30 µM PPADS in the recording chamber had no effect on IJPs evoked by distension when local transmission from distension sensitive intrinsic sensory neurons was blocked by low Ca^2+^, high Mg^2+^ in the stimulation chamber. Under these conditions, activity can only be carried into the recording chamber by anally directed axons of distension sensitive intrinsic sensory neurons, so P2Y receptors are probably not involved in transmission from these neurons to neurons in the recording chamber. This adds to earlier data indicating that nicotinic and muscarinic acetylcholine, P2X and 5-HT_3_ receptors are not involved in transmission at these synapses [11]. Similarly, NK_1_ and NK_3_ tachykinin receptors do not appear to be involved, although the former can facilitate P2X-mediated transmission from interneurons to inhibitory motor neurons [10].

Another contributor to the descending inhibitory pathway needs to be considered, these are long anally projecting inhibitory motor neurons identified in both retrograde tracing and electrophysiological studies [18], [26]. These neurons project far enough that neurons with cell bodies in the central chamber have terminals in the recording chamber. Blockade of synaptic transmission in the central chamber when distension is applied in the oral chamber would prevent descending axons of intrinsic sensory neurons exciting long inhibitory motor neurons, which may account for the difference in efficacy of high concentrations of PPADS and the low Ca^2+^, high Mg^2+^. The failure of PPADS together with hexamethonium and granisetron to block these synapses is consistent with previous findings that transmission from intrinsic sensory neurons to inhibitory motor neurons is insensitive to blockers of all receptors known to mediate fast EPSPs in guinea pig enteric nervous system [11]. Thus, transmission to NOS-descending interneurons from distension activated descending inhibitory neural pathways is primarily via P2Y_1_ mediated slow EPSPs with little role for fast EPSPs. These are the only neurons with such slow EPSPs, so the effects of PPADS and MRS2179 in the central chamber on descending inhibitory reflexes evoked from the oral chamber can only be by blocking transmission to these neurons.

### The endogenous ligand mediating distension evoked slow EPSPs

It is often assumed that ATP is acting at P2 receptors to mediate synaptic potentials (e.g. [27], [28], but ADP also activates P2Y_1_ receptors with various reports that ADP is more potent, less potent or roughly equipotent to ATP (for review [29]). Recent studies have implicated an alternative purine, β-nicotinamide adenine dinucleotide (β-NAD) as a transmitter acting at P2Y_1_ receptors in inhibitory neuromuscular transmission in mouse, human and non-human primate gut [30], [31]. A key element of this argument is that the muscle's response to ATP is not blocked by PPADS or a specific P2Y_1_ antagonist, while those to β-NAD are. However, we found that depolarizations evoked by ATP are blocked by PPADS in neurons with and without distension evoked slow EPSPs. Thus, β-NAD, ATP and ADP are all viable candidates for the transmitter producing P2Y_1_ mediated slow EPSPs in NOS descending interneurons in guinea-pig ileum.

### Implications of physiological excitatory transmission via P2Y_1_ receptors

It is well established that P2Y_1_ receptors mediate slow EPSPs evoked by electrical stimulation in myenteric and submucosal enteric neurons [13], [32], [33], [34], but the physiological significance of slow EPSPs in general has been difficult to determine. It is difficult to unambiguously identify a single site where antagonists of receptors mediating slow EPSPs act within a physiological pathway. P2Y_1_ receptors mediate IJPs in circular muscle [27], [28], [35], so antagonists will act on at least two different sites in the descending inhibitory pathway, unless a multichambered organ bath is used. Further, unless a physiologically relevant stimulus evokes both a pharmacologically distinct slow EPSP and an end organ response, it is almost impossible to discriminate between primary transmitter and modulatory roles for a receptor producing slow EPSP-like responses.

Although roles for P2Y_1_ [32], [33]. 5-HT_7_ [17], mGluR1 [34]and both NK_1_ and NK_3_ tachykinin receptors [12], [16], [36], [37]}[13] in electrically evoked slow EPSPs in myenteric or submucosal neurons have been identified, the other elements needed to establish physiological roles for these receptors are absent. Thus, while the present study gives some confidence to the idea that enteric slow EPSPs in general have major roles in transmission within physiological pathways, the specifics of these roles are unknown.

The present results also raise the question of the roles of P2Y_1_ receptors in the central nervous system, and more generally of metabotropic receptors that produce depolarizations of central neurons when activated. It is usually assumed that P2Y_1_ receptors have a modulatory role in transmission, largely based on their localization on glial cells [38], [39], but there is evidence that they can be involved in direct excitation of central neurons [38], [40]. Similarly, mGluR1 receptors are important in regulating synaptic plasticity [41], [42], but their role in primary transmission remains unexplored, although they mediate slow depolarizations in some central neurons [43].

### In Conclusion

This study provides the first direct evidence that slow EPSPs mediated by P2Y_1_ receptors play the primary role in excitatory transmission at a physiologically defined synapse. These slow EPSPs are the primary response evoked in NOS-immunoreactive descending interneurons by distension, result from activation of more orally placed interneurons, are abolished by the P2 receptor antagonist PPADS and mimicked by ATP applied to the same neurons. PPADS reduces transmission of distension-evoked descending inhibition through the intermediate chamber of a three chambered organ bath, where the antagonist would be acting primarily on interneuron to interneuron synapses and on transmission from intrinsic sensory neurons to inhibitory motor neurons. Transmission at the latter type of synapse and at synapses between intrinsic sensory neurons and descending interneurons in this pathway does not involve P2Y_1_ receptors, P2X receptors, nicotinic acetylcholine receptors or 5-HT_3_ receptors. The results suggest that slow EPSPs and their physiological roles may be a fertile area of research into the functions of metabotropic receptors known to be important in a variety of behaviours regulated by the brain.

## Materials and Methods

The methods used in this study have all been described previously [10], [11], [12].

Briefly, guinea-pigs (180–280 g) of either sex were killed by a blow to the back of the head, followed by severing the carotid arteries and spinal cord. The University of Melbourne Animal Experimentation Ethics Committee approved this procedure in accordance with the guidelines of the National Health and Medical Research Council of Australia. Segments of ileum (45 mm long) were removed 10 to 30 cm proximal to the ileocecal junction, flushed free of intestinal contents and placed into physiological saline (composition in mM: NaCl 118, KCl 4.8, NaH_2_PO_4_ 1, NaHCO_3_ 25, MgSO_4_ 1.2, glucose 11, CaCl_2_ 2.5, bubbled with 95%O_2_, 5% CO_2_). Nicardipine (1–2 μM) and hyoscine (1 μM) were added to the bathing solution to minimise myogenic and neurogenic contractions, respectively. This also has the effect of depressing descending excitation [20], so that the distension (see below) predominantly activated descending inhibitory reflexes. The segment was then opened along its mesenteric border and pinned flat, mucosa uppermost in a dissecting dish and prepared for either neuronal or circular muscle recording.

### Neuron preparation

The mucosa, submucosa and circular muscle were removed in the most anal 10 mm of the segment, revealing the myenteric plexus and longitudinal muscle, with the oral (35 mm) segment undisturbed. The preparation was transferred to an organ bath divided by a perspex/silicon partition (sealed with silicon grease) into two chambers at the junction of the intact tissue and the cleared myenteric plexus. Distending stimuli were applied in the oral chamber and intracellular recordings were made in the anal chamber [12]. These chambers were separately perfused at 2 ml/min with physiological saline at 35°C. To reduce the possibility of a distension having direct mechanical effects in the recording chamber, the ileum immediately oral to the partition was left slack so that increased tension produced by the distension was taken up in this region. The preparation was left to equilibrate for at least 1 hr before recordings were attempted.

Some experiments were performed in completely stripped preparations of myenteric plexus-longitudinal muscle. In these studies, circumferentially directed internodal strands supplying the ganglion containing the impaled neurons were stimulated via tungsten microelectrode positioned just above the preparation.

### Smooth muscle preparation

A 5 cm open segment of ileum was transferred mucosal-side up to an organ bath divided by perspex partitions into three chambers, each sealed with silicon grease and perfused separately with physiological saline at 35°C at 5 ml/min. Distending stimuli were applied either from the central or the most oral chamber with slack zones between the central and recording chambers, the distances between the centres of the two distending balloons and middle balloon and the recording site were each 15 mm. The ileum in the recording chamber was folded over so that the serosa was uppermost and in experiments using PPADS, the circular muscle was impaled through the serosal surface using methods described previously [11]. In the experiments using MRS 2179, recordings were made from the circular muscle using a flexibly mounted suction electrode (tip diameter 0.88 mm) attached to the serosal surface, which preferentially records circular muscle activity [44].

### Drugs

Drugs used in these experiments were nicardipine, adenosine tri-phosphate (ATP), 2-methyl-thio-ATP (2-MeSATP), serotonin (5-HT), hyoscine, hexamethonium, pyridoxal-phosphate-6-azophenyl-2′,4′-disulphonic acid (PPADS), all from Sigma Aldrich Fine Chemicals (Sydney, Australia), MRS 2179 from Tocris (Australian Laboratory Services, Sydney, Australia), tetrodotoxin (TTX) from Alomone, Jomar Diagnostics (Adelaide, Australia), SR 140333 and SR 142801 (gifts from Dr Emonds-Alt, Sanofi Recherche, France) and granisetron, a gift from SmithKline Beecham, Australia. All antagonists were made up as stock solutions in distilled water, usually at a concentration at least 1000 times that used in experiments, and then stored at 4°C until used. During experiments, appropriate aliquots of antagonists were diluted with the physiological saline to relevant final concentrations, which were then delivered to the organ baths via the superfusion system. Agonists (ATP and 2-MeSATP) were delivered to the cell bodies of impaled neurons via pressure ejection (duration 1 ms) from micropipettes (tip diameter 200 μm) using a DAD12 pressure application system (Adams and List, Westbury, NY, USA). In each case, the agonist concentration in the micropipette was 100 µM in Hepes buffered physiological saline.

### Quantitative analysis

Electrophysiological data were initially digitized and then stored on personal computers for subsequent measurement and analysis via either Axoscope 8 (Axon Instruments, Burlingame CA, USA) or AcqKnowledge 2.4 (SDR Clinical Technology, New South Wales, Australia). Inhibitory junction potentials in the descending reflex pathway are often biphasic (e.g. [10]). In these experiments, the amplitude of the initial peak was measured. All data in the text is reported as mean ± standard error of the mean, unless otherwise reported. Where appropriate washout of antagonists could be confirmed, data were analysed by the use of a one way analysis of variance (ANOVA). Where washout could not be confirmed, data were compared using paired t tests. Comparisons between different groups were made using unpaired t tests. In all cases, a *P* value <0.05 was taken to indicate statistical significance.
